# In vitro effects of lonidamine and 6-aminonicotinamide against *Echinococcus granulosussensu stricto* and *Echinococcus multilocularis*

**DOI:** 10.1186/s13567-020-00744-6

**Published:** 2020-02-26

**Authors:** Qi Xin, Miaomiao Yuan, Huanping Li, Xiaoxia Song, Jun Lu, Tao Jing

**Affiliations:** 1 Institute of Pathogenic Biology, School of Basic Medical Sciences, Lanzhou University, Lanzhou, China; 2 The Eighth Affiliated Hospital, Sun Yat-sen University, Shenzhen, China

## Abstract

Echinococcosis is a zoonotic disease caused by cestode species of the genus *Echinococcus*, which demonstrates considerable medical and veterinary concerns. The development of novel drugs for echinococcosis treatment is urgently needed. In this study, we demonstrated that lonidamine (LND) and 6-aminonicotinamide (6-AN) exhibited considerable in vitro effects against both larval- and adult-stage of *E. granulosussensu stricto (s. s.)* and *E. multilocularis*. The combination of LND and 6-AN exhibited a significantly higher activity than the single drug treatment. These results highlight the therapeutic potential of LND, 6-AN and the combination of LND and 6-AN for the treatment of echinococcosis.

## Introduction, methods and results

Echinococcosis, acquired by the infection with the larval stage of the genus *Echinococcus*, is a severe and life-threatening zoonotic disease. Cystic echinococcosis (CE) is caused by *Echinococcus granulosussensu lato (s. l.)* and alveolar echinococcosis (AE) by *Echinococcus multilocularis*. The adult tapeworms inhabit in the intestine of the definitive hosts (mainly dogs for *E. granulosuss. l.* and foxes and dogs for *E. multilocularis*) and both diseases are acquired through the accidental ingestion of parasite eggs shed by definitive hosts. The parasites often result in organ dysfunction or even failure in humans and domestic livestock, causing severe public health problem and economic loss.

Albendazole (ABZ) is the currently used drug against echinococcosis, however, it only shows about 30% of cure rate against CE [1]. As ABZ only has parasitostatic but not parasitocidal effect in AE patients, the patients must take the drug for a lifelong time to avoid recurrences and spread of metacestodes tissue [2], which unavoidably results in many adverse effects, such as nausea, hepatotoxicity and neutropenia. Therefore, it is necessary to identify novel potential chemotherapeutical treatment options for echinococcosis.

In *E. multilocularis* infection, metacestodes exhibit typical biological characteristics, such as progressive tumor-like growth and metastasize to other organs. Therefore, it is a useful and economical strategy to identify novel chemotherapeutics against *Echinococcus* by investigating the drugs inhibiting cancer cells proliferation [3]. A number of antitumor drugs, including 2-methoxyestradiol, imatinib, doxorubicin, cyclosporine, isoflavone genistein, rapamycin, bortezomib, tamoxifen, and 5-fluorouracil have been investigated for the treatment of echinococcosis. Lonidamine (LND) is a derivative of indazole-3-carboxylic acid, and exhibits effects against many cancer cell lines [4]. Moreover, this agent has gone through a multitude of clinical trials as a single agent or in combination with other anticancer chemotherapeutics (such as cisplatin, diazepam, and paclitaxel) [5]. 6-aminonicotinamide (6-AN), an analogue of nicotinamide, exhibits anticancer activity [6], and particularly, when used in combination with other anticancer agents, sensitizes cancer cells to those agents [7, 8]. Therefore, we speculated that LND and 6-AN could exert effective activities against parasitic helminthes, including *Echinococcus*. In this study, we investigated the in vitro effects of LND, 6-AN and the combination of LND and 6-AN against *E. granulosussensu stricto (s. s.)* and *E. multilocularis*.

**Figure 1:**
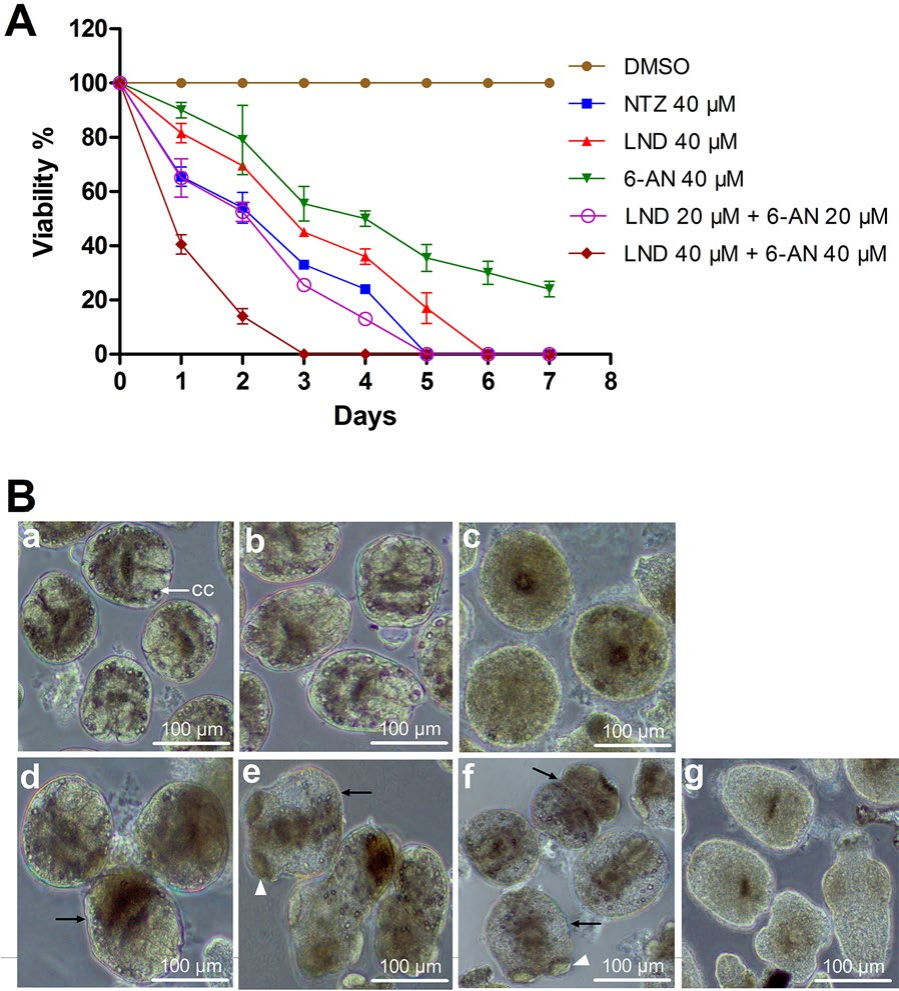
**In vitro efficacy of the drugs against*E. granulosus s. s.*protoscoleces**. **A** Survival of protoscoleces after treatment with LND, 6-AN, and their combination. **B** Morphologies of protoscoleces after 3 days of treatment with LND, 6-AN, and their combination. **a** Untreated protoscoleces. White arrow points towards calcareus corpuscles (cc); **b** Protoscoleces in culture medium containing 0.2% DMSO; **c**, **d**, and **e** Protoscoleces incubated with 40 μM NTZ, 40 μM 6-AN and 40 μM LND, respectively; **f** Protoscoleces incubated with 20 μM LND + 20 μM 6-AN. Note the distortion and vesiculation of protoscoleces (black arrow), the collapse of suckers (white arrowhead) and the decrease of calcareus corpuscles; **g** Protoscoleces incubated with 40 μM LND + 40 μM 6-AN.

*Echinococcus granulosus s. s.* protoscoleces were isolated aseptically from hydatid cysts in the liver of naturally infected sheep from a slaughter house in Xining, Qinghai, China. One hundred protoscoleces per well were cultured in 1 mL RPMI 1640 culture medium (supplemented with 12 mM HEPES, 2 mM glutamine, 100 U/mL penicillin, and 100 μg/mL streptomycin) without phenol red. The drugs were added to the cultures at the following concentrations: 40 μM nitazoxanide (NTZ), 40 μM LND, 40 μM 6-AN, 20 μM LND + 20 μM 6-AN and 40 μM LND + 40 μM 6-AN. Protoscoleces incubated in culture medium containing 0.2% dimethyl sulfoxide (DMSO) served as a control. Protoscoleces were observed microscopically every day and viability was evaluated by trypan blue exclusion test [9]. Each treatment was performed in duplicate and experiments were repeated twice. As positive control, NTZ killed 75% protoscoleces after 4 days of treatment, and 100% after 5 days (Figure 1A). Comparatively, 6-AN killed 65% and 76% protoscoleces, after 5 and 7 days of treatment, respectively, while LND killed 82% protoscoleces after 5 days and 100% protoscoleces after 6 days. The results demonstrated that LND exhibited higher efficacy than 6-AN, but slightly less than did NTZ. When 40 μM LND and 40 μM 6-AN were applied in combination, the efficacy was markedly increased, with 86% of protoscoleces killed after 2 days of treatment and 100% after 3 days. The combination of 20 μM LND + 20 μM 6-AN resulted in an eradication of 100% protoscoleces after 5 days of treatment. This effect was stronger than 40 μM LND or 40 μM 6-AN alone as well as NTZ (Figure 1A), and fully demonstrated the significant synergism of the combination. The morphological changes of protoscoleces after a 3-day treatment with LND, 6-AN or their combination further confirmed their efficacy (Figure 1B). Compared with control protoscoleces, most protoscoleces treated with LND or 6-AN exhibited distinct alterations, such as distortion and vesiculation, collapse of suckers, and decrease of calcareus corpuscles. Moreover, the combination of LND and 6-AN induced more severe damages than that did both drugs alone, with apparent destruction of parenchyma.

**Figure 2:**
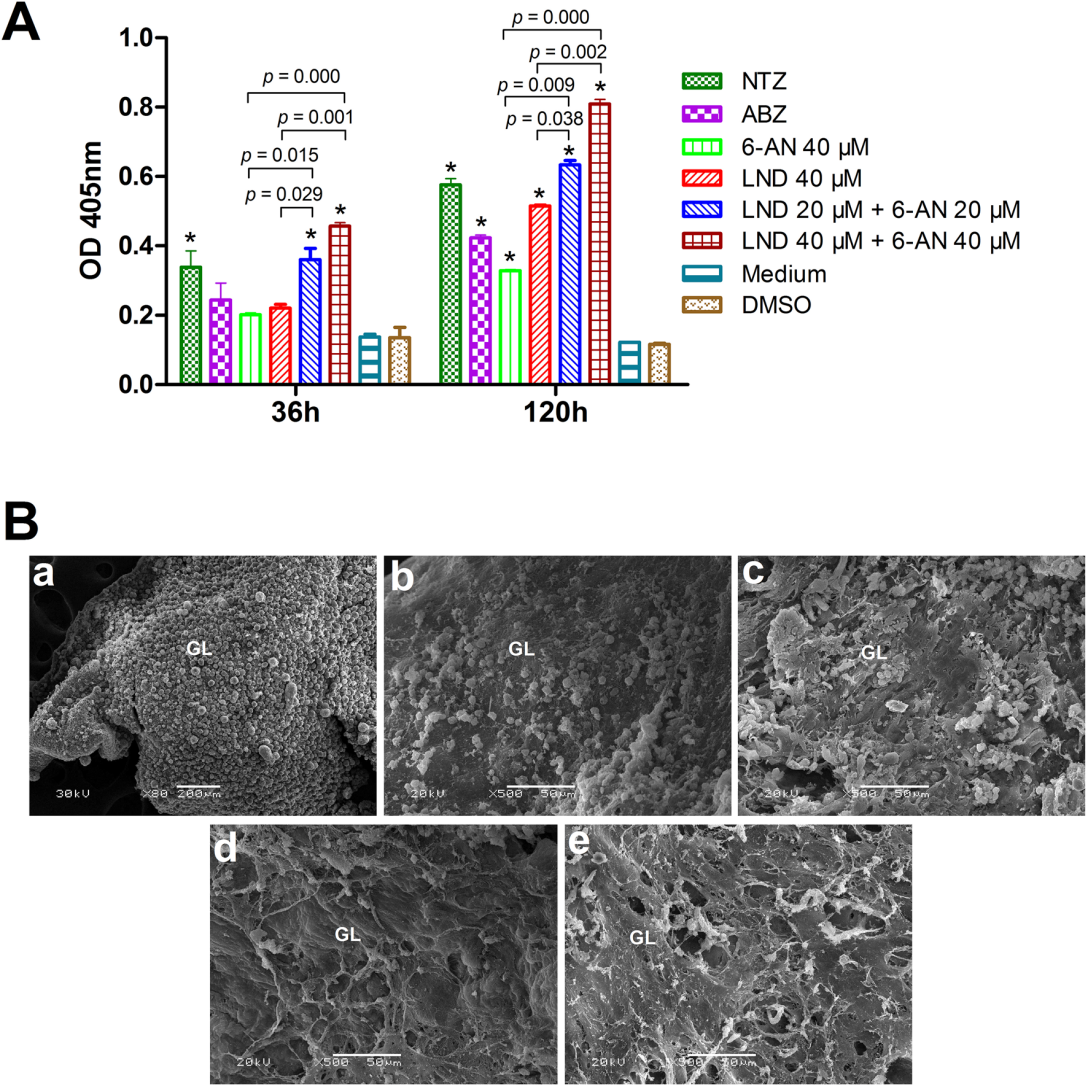
**In vitro efficacy of the drugs against*E. multilocularis*metacestodes**. **A** The release of alkaline phosphatase activity from *E. multilocularis* metacestodes (*Em*AP) after treatment with LND, 6-AN, and their combination. *p < 0.05 vs. DMSO control. **B** Morphologies of metacestodes after 5 days of treatment with LND, 6-AN, and their combination. **a** Metacestodes in culture medium containing 0.2% DMSO. Note the typical morphology of the germinal cells. GL, germinal layer; **b**–**e** Metacestodes treated with 40 μM 6-AN, 40 μM LND, 20 μM LND + 20 μM 6-AN and 40 μM LND + 40 μM 6-AN, respectively.

To evaluate the activities of the drugs against *E. multilocularis* metacestodes (isolate Xinjing), the metacestodes were dissected from experimentally infected Mongolian gerbils (*Meriones unguiculatus*) and the in vitro culture of metacestodes was performed, as previously described [10]. The drug concentrations used were the same as above. After 36 and 120 h of incubation, culture supernatants were collected for measurement of *E. multilocularis* alkaline phosphatase (*Em*AP) activity, as previously described [11]. The experiments were performed in triplicate and repeated thrice. The results are presented as the mean ± standard deviation and analyzed by One-way ANOVA using SPSS 19.0 software. After 36 h of treatment, LND and 6-AN both induced an increased release of *Em*AP activity in culture supernatants (Figure 2A), which indicated a drug-induced damage of vesicle cells from *E. multilocularis* metacestodes. The *Em*AP activities were strongly increased after 120 h of treatment, demonstrating the time-dependent effects of the drugs on the vesicles. Moreover, LND induced a significant increase in *Em*AP activity compared with ABZ treatment. There was no significant difference by comparison with NTZ treatment. For similar treatment period, 20 μM LND + 20 μM 6-AN resulted in a higher release of *Em*AP than did 40 μM LND, 40 μM 6-AN, and 40 μM ABZ. In addition, 40 μM LND + 40 μM 6-AN induced a significant increase in *Em*AP activity than the other groups, including NTZ treatment. Such increase in *Em*AP activity coincided with the morphological alterations observed by scanning electron microscopy (SEM) (Figure 2B). The metacestodes treated with 6-AN or LND exhibited noticeable damages, including the detachment of the germinal layer (GL) cells and the residual cellular materials in the GL. In comparison to 6-AN, LND treatment resulted in more stronger damages on metacestodes. Moreover, 20 μM LND + 20 μM 6-AN caused more severe damages than 40 μM LND or 40 μM 6-AN. The most pronounced damage on metacestodes was observed after 40 μM LND + 40 μM 6-AN treatment, with the loss of the cellular integrity of the GL and complete disintegration of the major portion of the GL tissues.

**Figure 3:**
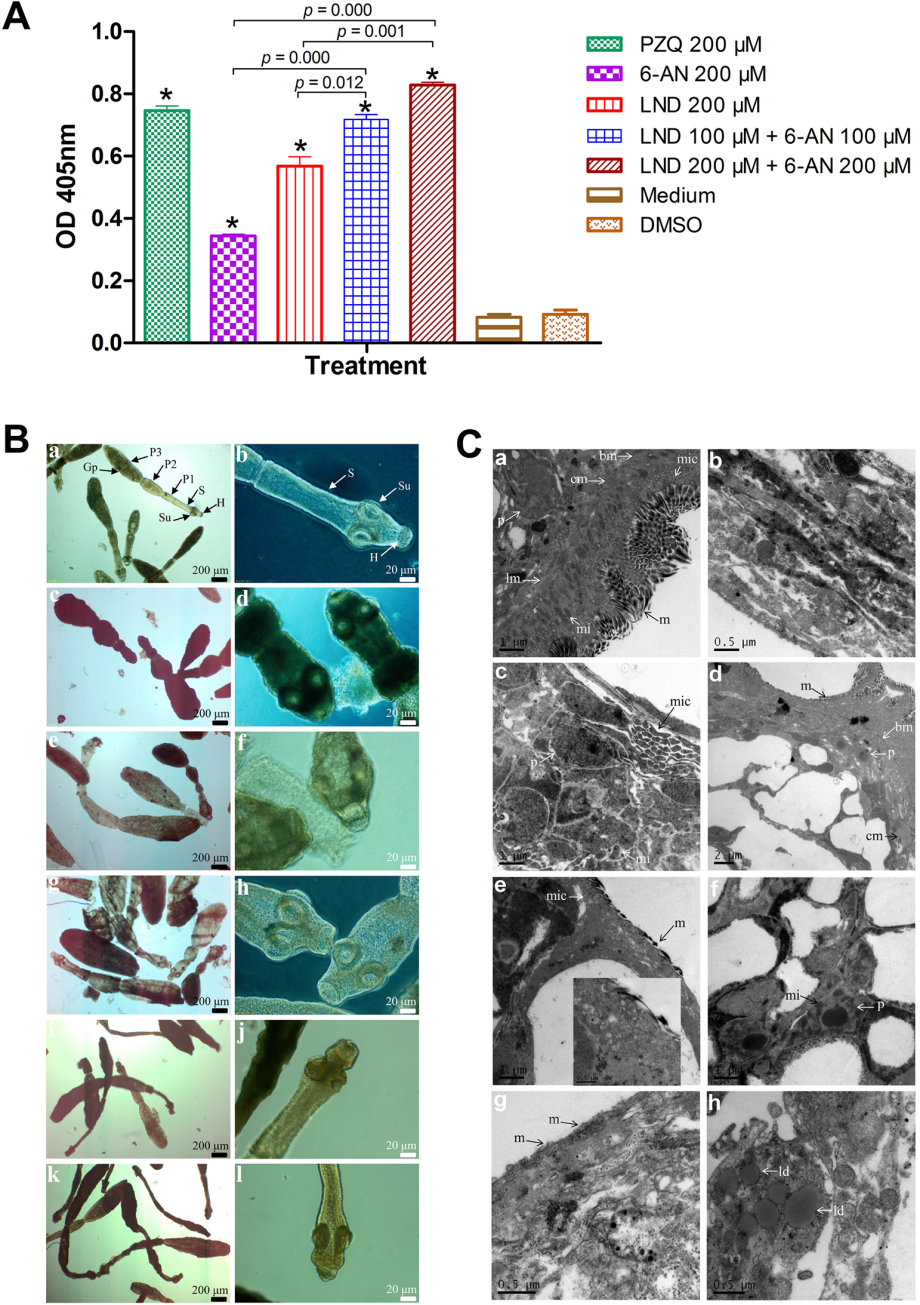
**In vitro efficacy of the drugs against adult*E. granulosus s. s.*A** The release of alkaline phosphatase from adult *E. granulosus s. s.* (*Eg*AP) after treatment with LND, 6-AN, and their combination. *p < 0.05 vs. DMSO control. **B** Morphologies of adult *E. granulosus s. s.* after treatment with LND, 6-AN, and their combination. **a**, **b** Tapeworms incubated in culture medium containing 0.2% DMSO. Note the concave suckers (Su) on the scolex (S), the intact first proglottid (P1), second proglottid (P2) and terminal proglottid (P3) with genital pore (Gp) as well as the complete hooks (H); **c**–**l** Tapeworms treated with 200 μM PZQ (**c**, **d**), 200 μM 6-AN (**e**, **f**), 200 μM LND (**g**, **h**), 100 μM LND + 100 μM 6-AN (**i**, **j**) and 200 μM LND + 200 μM 6-AN (**k**, **l**), respectively; **C** Ultrastructures of adult *E. granulosus s. s.* after 4 h of treatment with LND, 6-AN, and their combination. **a** Tapeworms incubated in culture medium containing 0.2% DMSO. Note the normal and distinct morphology (m, microtriches; mi, mitochondria; mic, microfilament; cm, circular muscle bundle; lm, longitudinal muscle bundle; bm, basement membrane; p, perikarya) and the large number and the length of microtriches; **b**–**h** Tapeworms treated with 200 μM PZQ (b), 200 μM 6-AN (**c**), 200 μM LND (**d**), 100 μM LND + 100 μM 6-AN (**e**, **f**) and 200 μM LND + 200 μM 6-AN (**g**, **g**), respectively. ld: lipid droplet.

Next, we evaluated the effects of LND, 6-AN and their combination against adult *E. granulosus s. s.*. Two beagle canines were orally infected with *E. granulosus s. s.* protoscoleces from hydatid cysts from naturally infected sheep. Thirty-three days after infection, adult *E. granulosus s. s.* were collected from the small intestine of euthanized dogs and 35 tapeworms were placed into 3 mL of RPMI 1640 culture medium without phenol red. Animal procedures and management were carried out in accordance with the protocols (2018-03-002) approved by the Institutional Animal Caring and Using Committee of Lanzhou University. The drugs were added to the cultures at the following concentrations: 200 μM praziquantel (PZQ), 200 μM LND, 200 μM 6-AN, 100 μM LND + 100 μM 6-AN and 200 μM LND + 200 μM 6-AN. After incubation for 4 h at 37 °C with 5% CO_2_, culture supernatants were collected for measurement of *E. granulosus s. s.* alkaline phosphatase (*Eg*AP) activity. Significant increases in *Eg*AP activity from culture supernatants were observed after treatment with LND, 6-AN, 100 μM LND + 100 μM 6-AN, 200 μM LND + 200 μM 6-AN and PZQ, in comparison to DMSO control (Figure 3A). LND induced a significant increase in *Eg*AP activity compared with 6-AN, while had no difference with PZQ. 100 μM LND + 100 μM 6-AN resulted in more significant increase in *Eg*AP activity than did 200 μM LND or 200 μM 6-AN. The most significant increase in *Eg*AP activity was induced by 200 μM LND + 200 μM 6-AN, in comparison to LND or 6-AN alone, and even PZQ. The morphological alterations of adult *E. granulosus s. s.* after treatment were consistent with the *Eg*AP activity assay. For optical microscopy (Figure 3B), tapeworms in DMSO control maintained a typical appearance: an integrated scolex composed of a rostellum, intact hooks and four concave suckers with a distinct neck and three proglottids. In comparison, 6-AN and LND treated tapeworms exhibited extensive alterations, including the dimming and distortion of body segments, partial loss of hooks on the rostellum, and contraction and distortion of the suckers. 100 μM LND + 100 μM 6-AN and 200 μM LND + 200 μM 6-AN both resulted in more pronounced changes than those induced both drugs alone. Noticeably, the morphological alterations caused by PZQ were quite different from those caused by LND and 6-AN, showing more starker dimming, swelling and shortening of the body segments (especially the first and second proglottid), destruction of the tegument, and severe distortion of the rostellum and suckers. Transmission electron microscopy (TEM) (Figure 3C) showed that the tapeworms from DMSO control had the intact and typical structure: the smooth and conical microtriches covered the intact tegument, round or oval microfilaments at the base of the microtriches, multiple mitochondrion presented within the distal cytoplasm and the basement membrane located at the bottom of the cytoplasm, the subcutaneous layer contained circular and longitudinal muscle bundles and perikarya. In comparison, 6-AN treated tapeworms exhibited partial shedding of microtriches and cellular destruction of perikarya. LND resulted in a marked reduction in the size and number of microtriches and destruction of the substantial portions of subcutaneous layer. 100 μM LND + 100 μM 6-AN resulted in more severe damages than did 200 μM LND or 200 μM 6-AN, with noticeable truncated and reduced microtriches and the residual perikarya which presented electron-dense and swollen mitochondrion. Moreover, 200 μM LND + 200 μM 6-AN resulted in the most severe damages, exhibiting severely damaged perikarya and amorphous organellar debris, and occurrence of lipid droplets. As observed by optical microscopy, PZQ resulted in different alterations from those induced by 6-AN and LND, with the outer covering microtriches completely disrupted.

## Discussion

This study investigates for the first time the in vitro effect of the anti-cancer drugs LND, 6-AN and the combination of LND and 6-AN against *E. granulosus s. s.* and *E. multilocularis*. LND and 6-AN both exhibited considerable efficacy against *E. granulosus s. s.* protoscoleces and *E. multilocularis* in a time dependent manner, particularly the efficacy of LND was more efficacious than that of ABZ, the current licensed drug for human echinococcosis. It is not surprising that LND has profound efficacy against *Echinococcus*, as it has exhibited a spectrum of antineoplastic activities [4, 5] and efficacy against several protozoan parasites, including *Leishmania mexicana* [12], *Trypanosoma cruzi* and *Trypanosoma brucei* [13]. However, the mechanism of action of LND against *Echinococcus* is not clear. Some studies reported that LND may interfere with cellular energy by inhibiting mitochondrial hexokinases [14] and mitochondrial electron transport [15] in cancer cells. Furthermore, the mechanism of the inhibition of *L. mexicana* by LND is through inhibition of energy metabolism [12] and for *T. brucei*, inhibition of hexokinase activity [16]. The previous studies revealed that although *E. granulosus s. s.* and *E. multilocularis* both have aerobic and anaerobic respiratory systems, the energy and intermediates production were mainly dependent on glycolysis [17, 18]. Thus, the interference with glycolysis by inhibiting hexokinase might be the mechanism of LND against *Echinococcus*.

LND produced a greater anti-*Echinococcus* effect than that observed with 6-AN. LND killed 100% of *E. granulosus s. s.* protoscoleces after 6 days of treatment. In contrast, 6-AN treatment killed 76% protoscoleces after 7 days (Figure 1A). In addition, on *E. multilocularis* metacestodes, LND resulted in 0.085 ± 0.002 and 0.400 ± 0.001 OD405 nm increase at 36 h and 120 h of treatment respectively compared with the DMSO group, higher than that with 6-AN (0.066 ± 0.002; 0.212 ± 0.001) (Figure 2A). Likewise, LND produced a higher increase in OD405 nm (0.477 ± 0.014) than did 6-AN (0.252 ± 0.004) on adult *E. granulosus s. s.* (Figure 3A). The morphological and ultrastructural alterations confirmed these effects (Figures 1B, 2B and 3B). As we known, in the past years, 6-AN, which is believed to be a competitive inhibitor of glucose-6-phosphate dehydrogenase (G6PD) [19, 20], could inhibit G6PD to catalyse the conversion of glucose-6-phosphate (G6P) to 6-phosphoglucono-δ-lactone (6PGδL), the first step of the pentose phosphate pathway (PPP) and has been used for chemotherapy of various tumors [6]. Therefore, the possible mechanism of 6-AN against *Echinococcus* might be through inhibiting the PPP, an alternate metabolic pathway involved in energy production and can generate NADPH and important metabolic intermediates (pentose-5-phosphate) for synthesis of macromolecules [21, 22].

In addition to the efficacy against the larval stage of *E. granulosus s. s.* and *E. multilocularis*, LND and 6-AN also exhibit significant effects on the adult stage of *E. granulosus s. s.* (Figure 3). As we known, the adult tapeworms inhabit in the intestine of the definitive hosts at a relative anaerobic environment and depend on glycolysis for energy and intermediates production [23], and thus, the adult tapeworms are sensitive to LND and 6-AN. Furthermore, as observed by optical microscopy and TEM, the morphological and ultrastructural alterations induced by LND and 6-AN were different from the changes in PZQ-treated parasite (Figure 3B). LND and 6-AN mainly induced destruction of substantial portions of subcutaneous layer but microtriches still remained in the tegument, while PZQ completely disrupted the outer covering microtriches and the integrity of tegument. The same alterations by PZQ have also been reported by Conder et al. [24]. The different changes probably correspond to the different mode of action of the drugs. PZQ treatment results in osmotic lytic alterations of tegument, the loss of organic material from tapeworm and thus leads to the death of parasites [24, 25]. In contrast, LND and 6-AN may interfere with glucose and respiratory metabolism, respectively.

Our results demonstrated that the combination of LND and 6-AN had stronger effect than that did both drugs alone. Moreover, the combination exhibited not only the best efficacy, but also the faster action. At the same final concentration, the combination killed 100% protoscoleces after 5 days of treatment, to be compared with 100% protoscoleces killed after 6 days of treatment with LND and 76% protoscoleces killed after 7 days treatment with 6-AN (Figure 1). This characteristic was confirmed by *Em*AP activity assay (Figure 2A), morphological and ultrastructural alterations. We suppose the enhanced effect by the combination might be due to the complementary disruption of biochemical pathways by LND and 6-AN. As a competitive G6PD inhibitor, 6-AN could suppress PPP reduce the cellular energy, and impair DNA synthesis and repair. Both LND and 6-AN induce depletion of metabolic energy, yet they target different sites in energy generation pathways. Their use in combination might enhance energy depletion, and thereby yield significant effect against *Echinococcus*. Therefore, it suggests that the inhibition of both pathways of energy production may achieve synergetic effect against *Echinococcus*. As we known, toxicity, weak efficacy, and especially rapidly increasing drug-resistance hinder the effectiveness of chemotherapy to numerous diseases including echinococcosis. Therefore, the combination of drugs is one of the solutions to the above problems, which has many advantages including the enhancement of efficacy and delay of drug resistance [26].

In conclusion, in the present study, we demonstrate that LND and 6-AN exhibit considerable effects against both larval- and adult-stage of *E. granulosus s. s.* and *E. multilocularis*. Furthermore, the combined treatments with LND and 6-AN exhibit stronger effects than that did both drugs alone. The present results demonstrate the therapeutic potential of LND, 6-AN, and particularly their combination against *E. granulosus s. s.* and *E. multilocularis*. Further studies will be performed by investigating the in vivo efficacy of LND, 6-AN and their combination against *Echinococcus* spp. as well as the mechanism of action.
